# Supplementary material for: Clinical Inference of Serum and Bone Sclerostin Levels in Patients with End-Stage Kidney Disease
Source: J Clin Med. 2019 Nov 20;8(12):2027. doi: 10.3390/jcm8122027 (PMC6947521; doi:10.3390/jcm8122027)
Supplement: Supplementary file 1 [file jcm-08-02027-s001.pdf]

Supplementary files

Supplementary Table S1. Bone histomorphometric parameters and serum markers of bone metabolism of the total ESKD cohort and according to the presence of diabetes mellitus.

	**ESKD Patients All**		**Diabetes (*n* = 14)**		**Non-Diabetes (*n* = 54)**		***p*-Value**
			**Histomorphometric bone parameters**				
**B.Ar (%)**	**22.11**	±6.44	21.59	±5.59	22.25	±6.69	0.9090
**Min.Area** (%)	98.3	(2.10)	98.0	±1.29	98.3	(2.24)	0.7219
**O.Ar** (%)	1.70	(2.09)	2.42	±1.22	1.60	(2.16)	0.2679
**O.Pm** (%)	19.92	(14.95)	19.67	±8.34	19.92	(16.52)	0.6110
**E.Pm** (%)	4.42	(4.92)	5.24	±3.59	4.41	(4.97)	0.9698
**O.Wi** (m)	7.408	(2.768)	8.208	±1.925	7.329	(2.954)	0.3249
**Ob.Pm(T)** (%)	1.33	(3.98)	2.70	±2.41	1.27	(3.87)	0.3510
**Oc.Pm(T)** (%)	0.63	(1.17)	0.34	(1.03)	0.67	(1.35)	0.4137
**Tb.Th** (m)	145.4	(42.0)	127.1	(37.0)	147.7	±25.5	0.2066
**Tb.N** (mm^{-1})	1.936	±0.4654	1.977	±0.4372	1.925	±0.476	0.7086
**Tb.Sp** (m)	379.7	(188.0)	379.6	(153.5)	379.7	(187.1)	0.7086
			**Serum markers of bone metabolism**				
**BsAP** (g/L)	19.95	(18.48)	35.71	±21.71	19.60	(14.62)	0.1393
**P1NP** (g/L)	83.65	(72.17)	91.44	± 63.02	88.35	(72.97)	0.5135
**TRAP5b** (U/L)	5.74	(3.63)	5.98	±3.04	5.93	±2.38	0.8513
**lnPTH** (pg/mL)	5.3	(1.1)	5.1	±1.1	5.3	(0.9)	0.4450
**FGF23** (pg/mL)	1434.0	(6806.8)	1225.0	(4532.3)	1665.0	(7701.1)	0.9701
**OPG** (ng/mL)	9.2	(6.8)	12.2	±4.8	8.9	(6.2)	0.1137
**sRANKL** (ng/mL)	0.087	(0.098)	0.086	(0.116)	0.087	(0.096)	0.9368

Values are presented as mean ± SD or median (IQR) when variables were not normally distributed (*n* = 68). * $p \leq 0.05$, ** $p \leq 0.01$. B.Ar: bone area (% of tissue area), Min.Ar; Mineralized bone area (% of bone area), O.Ar: osteoid area (% of bone area), O.Pm: osteoid perimeter, E.Pm: eroded perimeter, O.Wi: osteoid width, Ob.Pm(T): osteoblast perimeter (relative to the total perimeter), Oc.Pm(T): osteoclast perimeter (relative to the total perimeter), Tb.Th: trabecular thickness, Tb.N: trabecular number, Tb.Sp: trabecular spacing, BsAP: bone-specific alkaline phosphatase, P1NP: procollagen type 1 N-terminal propeptide, TRAP5b: Tartrate-resistant acid phosphatase 5b, lnPTH: natural logarithm parathyroid hormone, FGF23: fibroblast growth factor 23, OPG: osteoprotegerin, sRANKL: soluble receptor activator of nuclear factor kappa-B ligand.

Supplementary Table S2. Spearman correlation matrix of skeletal sclerostin expression vs. serum sclerostin levels.

	**Number of Sclerostin Positive Osteocytic Lacunae/m^2 Bone Area**	
	**Spearman *r***	***p*-value**
	**Serum sclerostin levels**	
**Tecomedical**	0.3040	0.0117 *
**DiaSorin**	0.3301	0.0060 **
**BioMedica**	0.2887	0.0170 *
**R&D**	0.2574	0.0341 *
